# How I do it - endoscopic endonasal approach for pituitary tumour

**DOI:** 10.1007/s00701-016-2916-z

**Published:** 2016-08-15

**Authors:** N. Phillips, P. Nix

**Affiliations:** Department of Neurosurgery, Leeds General Infirmary, Great George Street, Leeds, LS1 3EX UK

**Keywords:** Endoscopic, Endonasal, Transphenoidal, Pituitary, Adenoma

## Abstract

**Background:**

Endoscopic endonasal surgery to access the anterior skull base has evolved in many centres worldwide and provides a minimally invasive alternative, with better patient experience, to open techniques.

**Method:**

We present a basic approach to a midline lesion that is the fundamental starting point for wider access to the skull base.

**Conclusion:**

The nuances of this technique illustrated here have been developed in many centres to provide a safe procedure that has a low incidence of complications and excellent potential.

**Electronic supplementary material:**

The online version of this article (doi:10.1007/s00701-016-2916-z) contains supplementary material, which is available to authorized users.

## Introduction

### Relevant surgical anatomy

Neurosurgeons should be familiar with the relevant rhinological anatomy of the approach, - navigation is helpful.

**Fig. 1:**
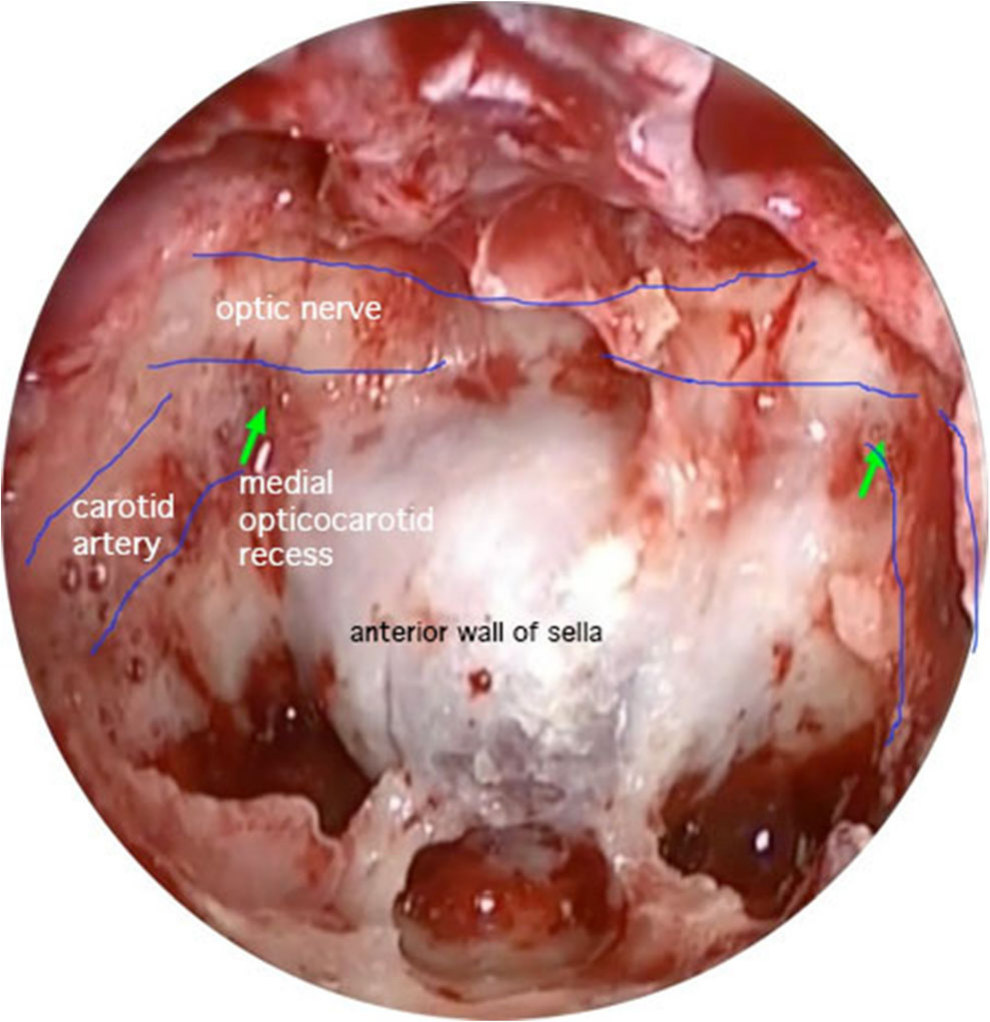
An endoscopic view showing important sphenoid sinus anatomy in the approach to a pituitary tumour

**Fig. 2:**
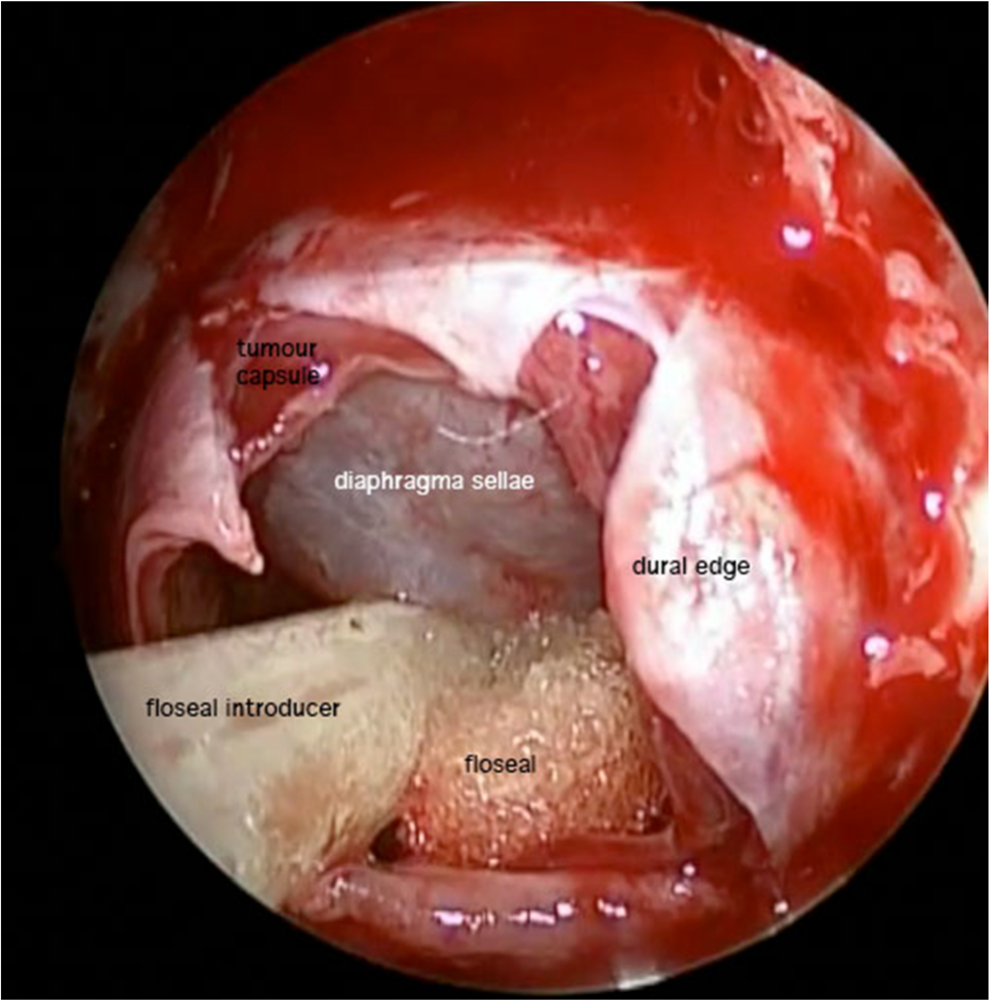
A view at the end of the procedure showing the tumour cavity and descent of the diaphragm sellae / tumour capsule

Major landmarks during the procedure are shown in Figs. 1 and 2.

### Description of the technique

#### Scans

MRI scans in 3 planes with and without contrast are performed preoperatively and examined for tumour size and lateral extension. A fine cut CT of the paranasal sinuses is examined for important variations such as non aerated sphenoid sinus, nasal deformity, and pattern of septation.

Electromagnetic navigation (Medtronic) is used routinely.

#### Anaesthesia

Stable anaesthesia with smooth emergence and reversal is essential to reduce coughing and postoperative haemorrhage.

#### Positioning

The patient is placed supine with the head elevated about 15°. Phenylephrine and lignocaine is used in the nose as a vasoconstrictor.

#### Technique

A zero degree endoscope is used by the second surgeon acting as cameraman.

If a large CSF leak is likely the thigh is draped for the harvesting of fascia lata.

Adrenaline (1:1000) soaked patties are used to achieve mucosal decongestant and vasoconstriction.

The septal mucosa is infiltrated with lignocaine 1 % with epinephrine 1/100,000. This will be the donor site for the nasoseptal flap.

We routinely remove the right middle turbinate and approach pituitary tumours through the right nostril when possible. The left middle turbinate is lateralised (outfractured) by gentle pressure with an elevator.

A nasoseptal flap [2] is routinely harvested on the right with its vascular pedicle containing the nasoseptal artery - a branch of the sphenopalatine artery. We are careful to stay below the olfactory epithelium.

We use the exact technique described by [2]. This reveals the sphenoid os on that side which is enlarged.

A generous posterior septectomy is performed to expose the vomer. When there is likelihood of intrasellar dissection wider exposure is required for the camera and instruments and this is achieved by a wide posterior ethmoidectomy. The bony opening over the dura extends to the medial carotids. Bony septa are drilled flat mindful that the median septum usually deviates laterally to overlie the carotid artery (See Fig. 1.)

The dural opening should allow as much intrasellar access laterally as possible. We use a X shaped incision.

As soon as the dura is opened tumour usually presents itself through the dural opening unless the target is a small adenoma. Tumour is biopsied using either a ring curette or Deckers rongeurs. The tumour is debulked using ring curettes, staying in the inferior half of the tumour to avoid breaching the diaphragm (or inverted dome of tumour capsule). The capsule is held up with a small neurosurgical patty to allow full exploration of the sella. The medial cavernous sinus is examined with a Doppler probe to ensure we have explored as far lateral as possible.

After tumour excision we inject Floseal (Baxter) into the sella. (see Fig. 2). We place the septal flap over the bony and dural defect, directly onto bone. Small amounts of tisseel (Baxter) anchor the flap in place which is also then wedged in position using Nasopore forte (Polyganics).

If there has been a persistent low volume csf leak during the procedure we use an additional inlay graft of a dural substitute (Duraform, Codman). If the leak is from a visible defect in the diaphragm we place a small piece of Spongistan (Ferrosan) over the defect. If there has been a high volume csf leak we would use fat in the sella and fascia lata as inlay and onlay grafts.

If there is persistent csf rhinorrhoea we would explore the operative site to examine the cause of the leak. We have not used lumbar drains or bed rest.

### Indications

The approach described here is a valuable initial step in approaching the anterior skull base for lesions based in the sella and suprasellar regions. It is flexible and versatile. It can reach from the frontal sinus to the odontoid peg. Modifications are described allowing access to more lateral areas, [1, 3].

This is our standard approach for pituitary adenomas, - secreting or non-secreting – microadenomas and macroadenomas. We are aware that some authors propose not harvesting the nasoseptal flap for small tumours requiring more limited access, or using for example the rescue flap [5].

### Limitations

Lesions such as meningioma and craniopharyngioma and pituitary adenoma that extend beyond the carotid artery laterally will not be accessible by this approach alone and require an extended, combined or cranial approach.

### How to avoid complications

The commonest complication of surgery around the sella is csf leak. We feel the best way to avoid this is by routinely using the nasoseptal flap [2] Our csf leak rate for non extended approaches is less than 1 % [4].

We try not to damage residual pituitary tissue to preserve pituitary function. Unless there is an obvious capsule we do not search for one as this can prejudice pituitary function.

The carotid artery is at risk during drilling. We only expose the carotid artery if necessary and identify it preoperatively on CT, and intraoperatively using navigation and doppler. In large tumours the carotid artery may be skeletonised by tumour expansion and extra care may be required during tumour excision.

Troublesome venous bleeding from sinuses is controlled with head elevation, compression with patties and Floseal (Baxter). The anterior intercavernous sinuses may bleed on opening the dura and can be pre-emptively clipped.

### Specific perioperative considerations

Examination of the pituitary dedicated MR and CT should identify abnormal anatomy. Careful endocrine assessment before during and after surgery is essential. A short period of corticosteroid support before formal endocrine assessment shortly after surgery is used. Careful assessment of fluid balance and management of diabetes insipidus is vital.

A routine postoperative scan and Goldman visual field tests are arranged for 3 months after surgery to assess residual tumour.

Patients are educated regarding csf leaks and given contact. They are advised to mobilise but to avoid blowing their nose, sneezing or coughing or straining if possible. They have an early (4 weeks) rhinological review. We warn patients that they will lose some sense of smell (and taste) temporarily.

### Summary - 10 key points

1. A team of neurosurgeon and ENT surgeon is recommended.
2. Examination of the MR and CT imaging is mandatory
3. Anaesthetic conditions should promote minimal blood loss during the approach.
4. Consider a nasoseptal flap in most cases
5. A generous posterior septectomy provides space for instrument manoeuvre.
6. The dural opening should allow wide access to the sella.
7. Explore the lateral walls of the sella and medial cavernous sinus.
8. Use a small Doppler probe to identify the carotid artery as the lateral limit of tumour excision
9. The diaphragma sellae can be held out of the operative field using a neurosurgical patty
10. Ensure the flap is placed on bone with mucosa removed.

## Electronic supplementary material

Below is the link to the electronic supplementary material.

ESM 1 (MOV 264119 kb)
